# Impact of faecal calprotectin measurement on clinical decision-making in patients with Crohn’s disease and ulcerative colitis

**DOI:** 10.1371/journal.pone.0223893

**Published:** 2019-10-24

**Authors:** Anna Luisa Bathe, Eirini Mavropoulou, Nicolae-Catalin Mechie, Golo Petzold, Volker Ellenrieder, Steffen Kunsch, Ahmad Amanzada

**Affiliations:** Department of Gastroenterology and Gastrointestinal Oncology, University Medical Center Goettingen, Goettingen, Lower Saxony, Germany; Grenoble Faculty of Medicine and Hospital, FRANCE

## Abstract

**Background:**

Faecal calprotectin (FC) seems to be the best available biomarker for the detection of intestinal inflammation in patients with inflammatory bowel disease (IBD). The aim of this study is to clarify whether the measurement of FC has changed the number of ultrasound and endoscopic procedures, drug modifications, as well as FC re-measurements in IBD patients.

**Methods:**

This retrospective study included 242 IBD patients with available FC values (case cohort) and 46 patients without an available FC value (control cohort). Clinical consequences such as carrying out abdominal ultrasound, endoscopy, drug modification or FC re-measurement at the next ambulatory presentation or during in-patient stay were collected. Statistical analysis was performed to determine the association between clinical decision-making and patient’s characteristics, especially FC value.

**Results:**

Overall, 192 (67%) clinical consequences were noted in both cohorts. In the case cohort 174 (91%) implications were noted compared to 18 (9%) in the control cohort (*P* < 0.001). In the case cohort, significantly more clinical consequences were detected in patients with Crohn’s disease (CD) as well as in ulcerative colitis (UC) patients with a FC value > 250 mg/Kg than in patients with a value of ≤ 250 mg/Kg. In CD patients with high FC values significantly increased numbers of abdominal ultrasounds, endoscopies and FC re-measurements were noted. In UC patients with high FC values significantly increased numbers of abdominal ultrasounds, drug modifications and FC re-measurements were noted.

**Conclusion:**

Measurement of FC may alter physician’s clinical decision-making in IBD patients beside other clinical and diagnostic parameters. Further prospective and survey studies are warranted to evaluate the influence of FC measurement in the daily clinical decision-making.

## Introduction

Inflammatory bowel diseases (IBD) such as Crohn’s disease (CD) and ulcerative colitis (UC), are chronic diseases characterized by relapsing-remitting inflammation of the gastrointestinal tract [1, 2]. The aetiology of IBD is still unknown although the incidence and prevalence is increasing worldwide [3, 4]. Symptoms of both CD and UC are heterogeneous and unspecific [3, 5, 6]. Typically symptoms shown are chronic diarrhoea and abdominal pain [3, 5, 6]. The diagnosis is made on the basis of clinical features, endoscopic, histological and laboratory findings as well as cross-sectional imaging techniques such as computer tomography (CT), magnetic resonance imaging (MRI) and transabdominal ultrasound [3, 5–7].

Faecal calprotectin (FC) is a 36 kDa calcium- and zinc-binding protein first described in 1980 [4, 8, 9]. It represents 60% of cytosolic proteins in granulocytes and is directly proportional to neutrophil migration to the gastrointestinal mucosa [4]. Furthermore, FC is closely correlated with faecal excretion of ^111^indium-labelled leucocytes [10]. FC is easy to assess by enzyme-linked-immunosorbent assay (ELISA) and represents a non-invasive, cheap and sensitive marker of intestinal inflammation [9, 11]. Besides, FC is stable in stools for up to 7 days at room temperature and resistant to degradation [12]. Many studies have shown that FC is significantly higher in patients with active IBD than in patients with remission [13–15]. In addition, FC correlates with the clinical, endoscopic and histological activity in IBD patients [11, 16, 17]. FC also correlates well with colonic intestinal inflammation in both CD and UC, but is less reliable in detecting small bowel inflammation in CD [18]. The main task in IBD is to predict relapse, to monitor disease activity, to assess treatment response and to evaluate the need for subsequent endoscopy [8, 19, 20]. To date FC seems to be the best available biomarker for the presence of intestinal inflammation [9, 16].

The aim of this study was to clarify whether the measurement of FC has changed the number of ultrasound examinations, endoscopies and drug modifications as well as FC re-measurements in patients with CD and UC. So far, there are hardly any statements or studies on the value of FC measurement with regard to sonographic, endoscopic and drug interventions.

## Methods

### Patient population

In this retrospective case control study, a total of 288 adult (≥ 18 years old) IBD patients with regular follow-up visits or in-patient stays at the University Medical Center Goettingen between January 2015 and December 2018 were enrolled in this study. Patients with CD were assigned to the CD group, patients with UC to the UC group. Patients with at least one FC measurement formed the case cohort, whereas all patients without FC measurements formed the control cohort. The reason for the absence of the FC value remains unclear due to the retrospective study design. Either the physicians did not arrange for FC measurement or the patients did not return their stool samples within 4 weeks after their appointment.

Inclusion criteria for the case cohort were: (1) Patients with CD or UC treated at the University Medical Center Goettingen, (2) Patients with at least one FC measurement, (3) Patients who returned their stool sample in less than 4 weeks after their appointment at the University Medical Center Goettingen, (4) Patients with regular follow-up visits or in-patient stays at the University Medical Center Goettingen, (5) Patients for whom the modified Harvey-Bradshaw index (mHBI) [21] or the partial Mayo score (pMS) [22] can be calculated and (6) Patients for whom the Montreal classification [23] can be determined.

Inclusion criteria for the control cohort were identical to the above, with patients having no FC measurements. In order to be assigned to the case or control cohort, all inclusion criteria had to be fulfilled.

### Data collection

The following parameters were taken from medical reports, our standardized IBD-questionnaires (including weight, height, body mass index, diagnosis, date of initial diagnosis, disease location, Montreal classification, stool frequency, stool consistency, stool admixtures, abdominal pain, active fistula, abscess, extraintestinal manifestations, mHBI, risk factors, family history, current and previous medications, surgery) and laboratory findings: (1) diagnosis of CD or UC, (2) age, (3) gender, (4) clinical activity using the mHBI or pMS, (5) Montreal classification, (6) FC levels in mg/Kg, (7) serum C-reactive protein (CRP) levels in mg/L, (8) platelets in 10^3^/μL, (9) disease duration, (10) previous intestinal resection (11) current therapy, (12) time between FC measurement or visit and consequence and (13) clinical consequences.

The serum concentration of CRP (normal < 5 mg/L) and platelet count (normal 150–350 x 10^3^/μL) were determined by utilizing the automated systems of the Central Laboratory of the Department of Clinical Chemistry at University Medical Center Goettingen. FC levels (normal < 50 mg/Kg) were measured with RIDASCREEN® Calprotectin according to the manufacturers’ instructions. Patients, for whom a FC measurement was indicated, were asked to bring a stool sample from their morning stool promptly after their appointment at the University Medical Center Goettingen or during their in-patient stay. The results of FC measurement of each individual patient were discussed in our medical team meeting. This team assessed at each patient’s next clinic visit whether clinical decision-making and ‘change in management’ was effected by FC testing. Both escalation of therapy, defined as an increased treatment dose or frequency, additional therapy or step-up in therapy, and de-escalation, defined as reduction in dose or frequency, step down or cessation of therapy, were counted as drug modification. Moreover, the clinical course was analysed for further investigations (including gastrointestinal ultrasound, gastroscopy or colonoscopy for disease activity assessment).

Local laboratory cut-off values were applied, with a result deemed ‘negative or normal’ if FC <50 mg/Kg, ‘positive’ if FC ≥50 mg/Kg and ‘definitely or highly positive’ if FC ≥250 mg/Kg. For this study, the cut-off value for FC was determined at 250 mg/Kg, as in other similar studies [11, 24–28]. FC > 250 mg/Kg was considered a positive value. Normally, it is standard practice to request a FC measure every three to six months at patient’s visit. Moreover, as recently suggested, the therapeutic goals for treat-to-target strategy for all IBD patients are to achieve clinical, endoscopic and laboratory remission [29]. In order to make the study clearer and more uniform only the first FC value was recorded per patient.

The mHBI for CD and pMS for UC were used as an instrument for determining the clinical disease activity. Clinical remission was defined by a mHBI < 5 points in CD and by a pMS < 2 points in UC patients. Disease location and behaviour were classified according to the Montreal classification.

In the case cohort all parameters, except the clinical consequences, were collected at the time of the first FC measurement. In patients of the control cohort the date of data collection was set to the date on which all parameters mentioned above, except FC were fully collected.

After all parameters were recorded in both cohorts, the clinical consequences at the next ambulatory presentation or during in-patient stay were analyzed. The following implications were recorded in this study: (1) absence of clinical consequence, (2) ultrasound examination, (3) endoscopy, (4) drug modification and (5) FC re-measurement (case cohort). In order to make the study clearer and more uniform only one clinical consequence at the next ambulatory presentation or during in-patient stay was recorded per patient. In this study drug modification was defined as an escalation or de-escalation of therapy. There was no distinction between escalation and de-escalation. An anonymized data set can be find under https://osf.io/x9s4h/.

### Statistical analysis

Firstly, a comparison was made between the case cohort and the control cohort, initially descriptive and then univariate. A multivariate analysis was not performed because there were only a few significant differences between the two groups. In addition, in both cohorts a separate statistical analysis for CD and UC was not possible due to the small number of patients in the control cohort.

Subsequently, the CD group was compared with the UC group, first descriptively and finally univariate. Only patients of the case cohort were included in the analysis. Again, we did not proceed to a multivariate analysis due to only one significant difference in the univariate analysis.

In a next step, comparisons were made separately for the CD group and for the UC group. Only patients of the case cohort were included in the analysis. Initially, the variables of the patients with a negative FC (≤ 250 mg/Kg) were compared with the variables of the patients with a positive FC (> 250 mg/Kg), first descriptively and finally univariate. After univariate analysis, a multivariate analysis was performed. A multivariate analysis for the single consequences was not possible because only one clinical consequence was recorded per patient.

Lastly, the group of patients with clinical consequences was compared with the group of patients with no clinical consequence, first descriptively and subsequently univariate. After univariate analysis, a multivariate analysis was performed.

Continuous variables were expressed as median and interquartile range (IQR), and categorical variables were expressed as percentage. The Mann-Whitney U test and Fisher’s exact test were used to compare non-parametric and dichotomous variables. All categorical variables with more than two forms were dichotomized for uni- and multivariate analysis. Clinical activity was classified as “Activity vs. Remission”. Clinical remission was defined by a mHBI < 5 points in CD and by a pMS < 2 points in UC patients. In accordance with the Montreal classification CD patients were dichotomized in “A1 vs. Not-A1”, “B1 vs. Not-B1” and “L1 vs. Not-L1”. UC patients were dichotomized in accordance with the Montreal classification in “E1 vs. Not-E1”.

After univariate analysis, a binary logistic regression analysis with the backward stepwise selection was performed. Results were expressed as odds ratio (OR) and 95% confidence interval (95%-CI). All variables with a *P* value < 0.1 in the univariate analysis were included in the logistic regression analysis model. All reported *P* values were two-sided, and *P* < 0.05 was considered as statistically significant. Statistical analysis was done using the SPSS 25 software for Mac OS (SPSS, Chicago, Illinois, USA).

### Ethical considerations

The study protocol was approved by the ethics committee of the University Medical Center of Goettingen (approval number 12/6/18). Individual consents from patients were not needed, because the study was retrospective. We fully anonymized the data before we accessed them and the ethics committee waived the requirement for informed consent.

## Results

**Fig 1:**
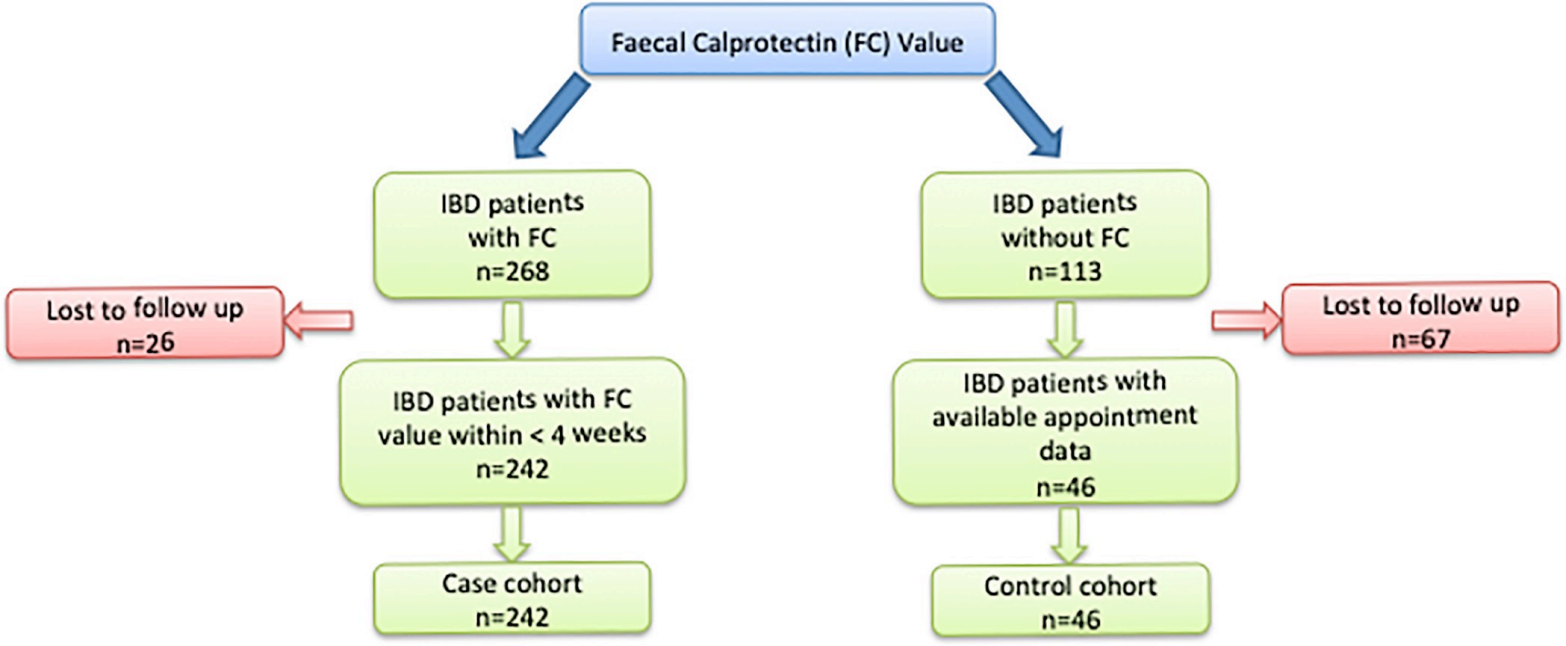
In- and exclusion of IBD patients dependent on the availability of a faecal calprotectin value within 4 weeks after their appointment.

The present study included 242 IBD patients with available FC values (case cohort) and 46 patients without FC measurements (control cohort). In the case cohort, 26 patients were lost to follow-up. In the control cohort most patients (n = 67) were excluded because there was no follow-up visit. Fig 1 shows an overview of the included and excluded patients of both cohorts. 16% (46/288) of IBD patients did not bring a stool sample for FC measurement or a FC measurement was not arranged.

### Basic characteristics

Table 1 shows the basic characteristics of the 288 included patients. Patients in the case cohort were significantly older than in the control cohort (*P* = 0.01; Table 1). Further evaluated variables such as gender, disease duration, status of disease activity, Montreal-classification, platelet counts, CRP and FC level as well as previous intestinal resection or current therapy did not significantly differ between both cohorts (Table 1).

**Table 1:** Basic characteristics of the IBD population.

Variable		Case cohort (n = 242)	Control cohort (n = 46)	p Value
**Median age, year (IQR)**		43 (25)	34 (23)	0.01
**Female Gender, n (%)**		130 (54)	24 (52)	0.87
**CD as IBD subtype, n (%)**		150 (62)	26 (57)	0.51
**mHBI, median (IQR)**		4 (5)	3 (4)	0.65
**pMS, median (IQR)**		1 (5)	2 (5)	0.54
**Median disease duration, year (IQR)**		7 (9)	6 ()	0.20
**Active disease, n (%)**		115 (48)	18 (39)	0.34
**Montreal-Classification, n (%)**	**A1**	20 (13)	5 (19)	0.54
	**Not-A1**	130 (87)	21 (81)	
	**L1**	28 (19)	2 (8)	0.26
	**Not-L1**	122 (81)	24 (92)	
	**B1**	58 (39)	12 (46)	0.52
	**Not-B1**	92 (61)	14 (54)	
	**E1**	6 (7)	1 (5)	1.00
	**Not-E1**	86 (93)	19 (95)	
**Median platelet counts, cells 10**^**3**^**/μL (IQR)**		280 (126)	284 (116)	0.53
**Median CRP level, mg/L (IQR)**		3.2 (8.1)	3.3 (14.4)	0.78
**Median Calprotectin level, mg/Kg (IQR)**		230 (1182)	n.a.	
**Previous intestinal resection, n (%)**		69 (29)	11 (24)	0.59
**Current therapy, n (%)**				
**No therapy**		30 (12)	8 (17)	0.32
**Aminosalicylate**		58 (24)	11 (24)	
**Corticosteroids**		14 (6)	6 (13)	
**Immunomodulator**		83 (34)	12 (26)	
**Biological**		57 (24)	9 (20)	
**Median time between FC measurement or visit** **and consequence, days (IQR)**		56 (61)	56 (63)	0.54

IQR: interquartile range; mHBI: modified Harvey-Bradshaw index; pMS: partial Mayo score; CRP: C-reactive protein; n.a.: not available; Immunomodulator: Azathioprine, Methotrexate, Tacrolimus; Biological: Infliximab, Adalimumab, Vedolizumab, Ustekinumab

57% (n = 26) patients of the control cohort had CD as IBD subtype. The median age and median disease duration of CD patients were 30 and 6 years, respectively. 50% (n = 13) of CD patients were female, 69% (n = 18) were in remission and 42% (n = 11) had an intestinal resection. 31% (n = 8) were not currently under a therapeutic regimen, 15% (n = 4), 12% (n = 3), 15% (n = 4) and 27% (n = 7) were treated with aminosalicylates, corticosteroids, immunomodulators and biologicals, respectively.

43% (n = 20) patients of the control cohort had UC as IBD subtype. The median age and median disease duration of UC patients were 37 and 5 years, respectively. 55% (n = 11) of UC patients were female and 50% (n = 10) were in remission. None of UC patients had an intestinal resection. 35% (n = 7), 15% (n = 3), 40% (n = 8) and 10% (n = 2) were treated with aminosalicylates, corticoidsteroids, immunomodulators and biologicals.

### Comparison of clinical consequences between case and control cohort

In the case cohort, a clinical consequence was reported in 174 (91%) patients at their next in- or out-patient visit. Significantly more implications were noted in the case cohort than in the control cohort (*P* < 0.001). In addition, significantly more ultrasound examinations (*P* = 0.01), endoscopies (*P* = 0.002) and drug modifications (*P* = 0.02) were performed in the case cohort compared to the control cohort (Table 2).

**Table 2:** Comparison of clinical consequences between case and control cohort with regard to FC availability.

	Consequence	Ultra-sound	Endo-scopy	Drug modification	Calprotectin re-measurement
**Overall, n (%)**	192 (67)	32 (11)	29 (10)	100 (35)	31 (11)
**Case cohort, n (%)**	174 (91)	30 (94)	28 (97)	85 (85)	31 (100)
**Control cohort, n (%)**	18 (9)	2 (6)	1 (3)	15 (15)	n.a.
**Case vs. Control cohort, *P* Value**	<0.001	0.01	0.002	0.02	n.a.

n.a.: not available

### Basic characteristics of the Crohn disease (CD) and ulcerative colitis (UC) group with available FC

Included CD patients with FC showed higher levels of CRP compared to UC patients (*P* = 0.004). Both groups differed significantly with regard to their medication at the time of FC measurement (*P* < 0.001). Age, gender, disease duration, platelet counts and calprotectin level did not differ between both groups (Table 3). FC level was significantly increased in male IBD patients as well as in IBD patients with active disease status (S1A and S1B Fig). Spearman correlations revealed that FC level were associated with age, CRP level and platelet count (S1C–S1E Fig).

**Table 3:** Comparison of Crohn’s disease (CD) and ulcerative colitis (UC) group with available FC.

Variable	CD group (n = 150)	UC group (n = 92)	*P* Value
**Median age, year (IQR)**	42 (25)	45 (21)	0.63
**Female Gender, n (%)**	86 (57)	44 (48)	0.18
**Median disease duration, year (IQR)**	8 (7)	6 (8)	0.08
**Active disease, n (%)**	70 (47)	45 (49)	0.79
**Median platelet counts, cells 10**^**3**^**/μL (IQR)**	276 (124)	287 (151)	0.60
**Median CRP level, mg/L (IQR)**	4.0 (8.5)	2.1 (3.8)	0.004
**Median Calprotectin level, mg/Kg (IQR)**	231 (1042)	203 (1932)	0.89
**Previous intestinal resection, n (%)**	69 (46)	0	n.a.
**Medication at time of FC measurement, n (%)**			
**No therapy**	28 (19)	2 (2)	<0.001
**Aminosalicylate**	13 (9)	45 (49)	
**Corticosteroids**	8 (5)	6 (7)	
**Immunomodulator**	59 (39)	24 (26)	
**Biological**	42 (28)	15 (16)	
**Median time between FC measurement** **and consequence, days (IQR)**	60 (56)	53 (78)	0.41

IQR: interquartile range; CRP: C-reactive protein; n.a.: not available; Immunomodulator: Azathioprine, Methotrexate, Tacrolimus; Biological: Infliximab, Adalimumab, Vedolizumab, Ustekinumab

### Comparison of clinical consequences between CD and UC group with available FC

In the CD group, a clinical consequence was carried out in 108 (62%) patients at their next visit. In the UC group, a consequence was reported in 66 (38%) patients. There were no significant differences in the number of implications between patients in the CD group and patients in the UC group (Table 4).

**Table 4:** Comparison of implications between the CD and UC group.

	Consequence	Ultra-sound	Endo-scopy	Drug modification	Calprotectin re-measurement
**Overall, n (%)**	174 (72)	30 (12)	28 (12)	85 (35)	31 (13)
**CD group, n (%)**	108 (62)	18 (60)	17 (61)	52 (61)	21 (68)
**UC group, n (%)**	66 (38)	12 (40)	11 (39)	33 (39)	10 (32)
**CD vs. UC group, *P* Value**	0.61	0.96	0.98	0.84	0.66

CD: Crohn’s disease; UC: ulcerative colitis

### Univariate and multivariate analysis of the CD group with regard to positive and negative FC results

After univariate analysis significant differences between patients with positive and patients with negative FC results were found in gender (*P* = 0.01), disease activity status (*P* = 0.03), age at diagnosis (*P* = 0.02), CRP level (*P* < 0.001), platelet count (*P* = 0.01) and the number of clinical consequences (*P* = 0.01). Thus, patients with a FC > 250 mg/Kg were more likely to be male, have an active disease status, were younger than 16 years old at diagnosis according to the Montreal classification, have an increased platelet count and elevated CRP level. In addition, significantly more implications were detected in this patient group than in patients with a FC value of ≤ 250 mg/Kg. A subanalysis of the individual consequences also revealed significant differences in the number of ultrasound examinations (*P* = 0.02), endoscopies (*P* = 0.001) and FC re-measurements (*P* = 0.02). Each of these consequences was found to be more frequent if FC result was > 250 mg/Kg. There was no significant difference in the number of drug modifications (*P* = 0.09).

After multivariate analysis for positive and negative FC, only CRP levels (*P* = 0.16) showed no significant difference. All other variables showed a statistically significant difference according to the univariate and multivariate analysis.

### Univariate and multivariate analysis of the CD group with regard to clinical consequences

After univariate analysis a comparison of variables between patients with and patients without clinical consequences revealed significant differences in CRP level (*P* = 0.04) and FC value (*P* = 0.01). Thus, both FC and CRP were significantly higher in the consequence-group than in the no-consequence-group.

After multivariate analysis, differences in FC (*P* = 0.91) and CRP (*P* = 0.05) diminished. All results are summarized in Table 5.

**Table 5:** Univariate and multivariate analysis with regard to calprotectin value and clinical consequences of CD group.

Variable	Calprotectin positive vs. negative			Consequence vs. No consequence		
	Univariate	Multivariate		Univariate	Multivariate	
	*P* Value	OR (95%-CI)	*P* Value	*P* Value	OR (95%-CI)	*P* Value
**Age**	0.67	n.a.	n.a.	0.21	n.a.	n.a.
**Gender**	0.01	0.27 (0.12–0.60)	0.001	0.47	n.a.	n.a.
**Activity vs. Remission**	0.03	2.53 (1.17–5.47)	0.02	0.37	n.a.	n.a.
**Montreal classification**						
**A1 vs. Not-A1**	0.02	6.05 (1.74–21.0)	0.01	0.11	n.a.	n.a.
**B1 vs. Not-B1**	0.74	n.a.	n.a.	0.58	n.a.	n.a.
**L1 vs. Not-L1**	0.31	n.a.	n.a.	1.00	n.a.	n.a.
**CRP**	<0.001	1.03 (0.99–1.06)	0.16	0.04	1.07 (1.01–1.13)	0.05
**Platelet count**	0.01	1.01 (1.01–1.10)	0.01	0.11	n.a.	n.a.
**Calprotectin**	n.a.	n.a.	n.a.	0.01	1.00 (1.00–1.00)	0.91
**Consequence (yes/no)**	0.01	4.80 (1.85–12.4)	0.001	n.a.	n.a.	n.a.

CD: Crohn’s disease; OR: odds ratio; 95%-CI: 95% confidence interval; CRP: C-reactive protein; n.a.: not available

### Univariate and multivariate analysis of the UC group with regard to positive and negative FC results

After univariate analysis significant differences between patients with positive and patients with negative FC results were found in age (*P* = 0.001), disease activity status (*P* < 0.001), CRP level (*P* = 0.004), platelet count (*P* = 0.01) and the number of clinical consequences (*P* < 0.001). Thus, patients with a FC > 250 mg/Kg were more likely to be younger, have an active disease status, increased platelet counts and elevated CRP levels. In addition, significantly more clinical consequences were noted in this patient group than in patients with a value of ≤ 250 mg/Kg. A subanalysis of the individual consequences also revealed significant differences in the number of ultrasound examinations (*P* = 0.03), drug modifications (*P* = 0.002) and FC re-measurements (*P* = 0.001). Each of these implications was found to be more frequent when FC values were > 250 mg/Kg. There was no significant difference in the number of endoscopies (*P* = 0.13).

After multivariate analysis for positive and negative FC, only CRP level (*P* = 0.33) and platelet count (*P* = 0.11) did not show a significant difference. All other variables showed a statistically significant difference according to the univariate and multivariate analysis.

### Univariate and multivariate analysis of the UC group with regard to clinical consequences

After univariate analysis a comparison of variables between patients with and patients without clinical consequences revealed significant differences in age (*P* = 0.02), gender (*P* = 0.02), CRP level (*P* = 0.01) and FC value (*P* < 0.001). Thus, patients with a clinical consequence were more likely to be younger, female and have increased FC values and elevated CRP level.

After multivariate analysis, there was still a significant difference in gender (*P* = 0.01) and FC values (*P* = 0.02). Age (*P* = 0.17) and CRP level (*P* = 0.11) did not significantly differ between patients with and patients without clinical consequences. All results are summarized in Table 6.

**Table 6:** Univariate and multivariate analysis with regard to calprotectin value and clinical consequences of UC group.

Variable	Calprotectin positive vs. negative			Consequence vs. No consequence		
	Univariate	Multivariate		Univariate	Multivariate	
	*P* Value	OR (95%-CI)	*P* Value	*P* Value	OR (95%-CI)	*P* Value
**Age**	0.001	0.95 (0.91–0.99)	0.01	0.02	0.97 (0.93–1.01)	0.17
**Gender**	0.54	n.a.	n.a.	0.02	5.21 (1.66–16.3)	0.01
**Activity vs. Remission**	<0.001	6.54 (2.26–18.9)	0.001	0.11	n.a.	n.a.
**Montreal classification**						
**E1 vs. Not-E1**	0.68	n.a.	n.a.	0.35	n.a.	n.a.
**CRP**	0.004	1.05 (0.95–1.16)	0.33	0.01	1.23 (0.95–1.59)	0.11
**Platelet count**	0.01	1.01 (0.99–1.01)	0.11	0.54	n.a.	n.a.
**Calprotectin**	n.a.	n.a.	n.a.	<0.001	1.01 (1.00–1.02)	0.02
**Consequence (yes/no)**	<0.001	0.26 (0.08–0.84)	0.03	n.a.	n.a.	n.a.

UC: ulcerative colitis; OR: odds ratio; 95%-CI: 95% confidence interval; CRP: C-reactive protein; n.a.: not available

## Discussion

Our study indicates that beside other clinical and diagnostic parameters FC measurement may have an influence on physician’s clinical decision-making in IBD patients. It is notable, that the case cohort (n = 242) was larger than the control cohort (n = 46). The measurement of FC therefore appears to be of great importance in the clinical routine of our clinic. Abej et al. [26] found that physicians trust the results of FC measurements. Huang et al. [24] described that physicians without knowledge of the level of FC made fewer decisions on the disease management and treatment. Another study of the Glasgow Royal Infirmary showed that the number of FC measurements increased from about 50 measurements per month in 2007 to more than 1000 measurements per month in 2012 [12]. This result also demonstrates the growing importance of FC in everyday clinical practice. In our study significantly more clinical consequences were detected in the case cohort compared to the control cohort, although the two groups did not differ with regards to the clinical parameters. FC therefore seems to have a major impact on clinical decision-making.

The univariate analysis of the individual consequences also showed significant differences in drug modifications, endoscopies and ultrasound examinations. More clinical consequences were detected in patients with FC measurements than in patients without. In a study by Rosenfeld et al. [25] it was shown that in 41 out of 243 patients an unplanned treatment change was made after FC measurement. Further studies confirmed the impact of FC measurements in the frequency of endoscopic procedures. In the study of Huang et al. [24] physicians without knowledge of the FC level would have performed endoscopy in 4 out of 36 patients. However, with knowledge of the FC level, physicians would have performed endoscopy in 16 of the 36 patients. Another study showed that 12% of pediatric patients with CD or UC underwent colonoscopy after FC measurement [27]. Our present study is in line with the above, with 12% (28/242) of our adult IBD cohort undergoing a colonoscopy. In addition, significantly more ultrasound examinations were performed in the case cohort compared to the control cohort. According to current knowledge, there are no published studies that have examined the impact of FC measurements on sonographic examinations.

A comparison between the CD and UC group showed no significant differences in the number of clinical consequences. Only CRP level was significantly higher in patients with CD than in patients with UC. This result is consistent with results from other studies [30, 31].

Our study showed that in CD patients with positive FC, significantly more clinical consequences were detected than in patients with negative FC. This result highlights the significant influence of FC on clinical decision-making in the CD group. This is also demonstrated by Rosenfeld et al. [25]. FC values > 250 μg/g resulted in significant more clinical consequences than values ≤ 250 μg/g. Another study showed that in 87% of all IBD patients with FC ≥ 250 μg/g the clinicians changed the therapy or ordered further examinations [26]. However, in both studies neither a separation between CD and UC nor a multivariate analysis to exclude confounding variables was performed.

In addition, our study demonstrated that FC values > 250 mg/Kg were associated with significantly more endoscopies than FC values ≤ 250 mg/Kg in patients with CD. These results are confirmed by current scientific literature. In two studies, 12 out of 14 or 21 out of 34 positive FC values (≥ 250 μg/g) resulted in colonoscopies [26, 27]. In addition, Motaganahalli et al. [11] showed that patients with positive FC (≥ 250 μg/mL) underwent colonoscopy earlier than patients with negative FC (< 100 μg/mL). However, none of these studies separated the CD from the UC cohort.

In our patients with CD a positive FC value resulted in significantly more ultrasound examinations. Ultrasound provides a feasible tool for the assessment of the disease activity with a sensitivity of 85% and a specificity of 91% and the location and extent of the disease with a sensitivity of 94% and a specificity of 86% [32, 33]. Ultrasound is particularly useful in the detection of complications in cases with a disease extension in the colon and terminal ileum [6, 33]. Possibly more ultrasound examinations were performed in patients with positive FC values to confirm the suspicion of active disease and possible complications.

A re-measurement of FC was performed significantly more often in CD patients with positive FC than in patients with negative FC. A review also states that a single measurement of a moderately elevated FC is not efficient for the clinical decision-making [34]. Furthermore, FC levels increase a few months before the appearance of symptoms of a relapse [2]. For this reason, regular FC measurements are useful in order to detect and treat an impending relapse early on.

The number of drug modifications in the CD group was not significantly different between patients with positive and negative FC results. Our data confirm the findings of Derwa et al. [28]. A FC value ≥ 250 μg/g was not associated with an escalation of the treatment in patients with CD. Therefore, a measurement of FC does not seem to influence treatment decision-making in patients with CD.

High FC values in the CD group showed a significant influence on clinical consequences after the univariate but not after the multivariate analysis. Therefore, FC seems to have an influence on clinical decision-making in patients with CD, but this influence is probably low after exclusion of all confounding variables. There are many studies that prove the validity of FC in CD. One study reported that FC values > 250 μg/g in patients with CD predict large ulcerations in the intestine with a sensitivity of 60.4% and a specificity of 79.5% [16]. Another study has shown that FC predicts relapses after surgical procedures for CD [35]. The CALM-study [36] also showed that a therapy escalation based on symptoms, a FC value ≥ 250 μg/g, a CRP ≥ 5 mg/L and a CDAI ≥ 150 leads more frequently to mucosal healing, deep, steroid-free and biological remission in CD patients than a therapy escalation based on symptoms alone. However, compared to UC, the validity of FC in CD is limited, especially in patients with isolated small bowel involvement [4, 7, 18–20].

In patients with UC and positive FC values (> 250 mg/kg) the uni- and multivariate analysis showed significantly more clinical decisions than in patients with negative FC. This may indicate a large influence of FC on clinical decision-making process in patients with UC.

UC patients with positive FC experienced significantly more drug modifications than patients with negative FC. Derwa et al. [28] came to the same conclusion. They reported that a positive FC value (≥ 250 μg/g) was associated with escalation of the therapy only in patients with UC, but not in patients with CD.

UC patients with positive FC received significantly more ultrasound examinations than patients with negative FC. Abdominal ultrasound is able to detect inflammation in the small and large intestine with a sensitivity of 80–90% [5]. Furthermore, ultrasound examinations offer the possibility to evaluate the response to treatment and predict the course of the disease in patients with UC [5]. The study of Parente et al. showed that ultrasound scores after three months of steroid therapy predicted the outcome of the disease at 15 months [37]. Therefore, physicians in our clinic perform more ultrasound examinations in patients with positive FC to confirm the suspicion of disease activity or non-response to therapy.

Additionally, in the UC group FC re-measurements were more frequently performed in patients with positive FC than in patients with negative FC. Current literature also recommends a repeated measurement of FC. De Vos et al. [38] demonstrated that two consecutive FC measurements with values above 300 mg/Kg predicts a relapse more accurately than a single measurement. Prager and Büning [39] also advise to carry out a FC measurement every three to six months.

There was no statistical difference in the number of endoscopies in UC patients with positive and negative FC. This may be explained by the fact that in UC FC correlates strongly with endoscopic scores and that correlation is also stronger in UC than in CD [16, 17, 40]. Therefore endoscopies were not needed for further decision-making.

High FC values in the UC group showed a significant influence on clinical consequences after the uni- and multivariate analysis, but the influence of FC after multivariate analysis seemed to be not very strong. However, the influence of FC on decision-making appeared to be stronger in patients with UC than in patients with CD. The superiority of the validity of FC in UC in comparison to CD is also proven by current literature [4, 7, 19, 20]. As already mentioned above, a strong correlation of FC with endoscopic scores for UC was shown in many publications [16, 17, 40]. This correlation seems to be stronger in UC than in CD [16, 17]. In addition, the level of FC can predict relapse and complete mucosal healing and differentiate between mild, moderate and severe activity [15, 20, 40]. Furthermore, FC predicts treatment response [41]. In contrast, Derwa et al. [28] have shown that the influence of FC on clinical investigations and therapy escalations seems to be low in patients with UC. Our present study is in line with the above.

The limitations of this study include the retrospective study design and the relatively small number of the included patients due to the separation between CD and UC. However, the total sample size is comparable with other similar studies. A further limitation is that in patients of the control cohort the reason for the absence of the FC value remains unclear due to the retrospective study design. In addition, it was not possible to determine the endoscopic disease activity because of the study’s retrospective design. Instead, disease activity was assessed by clinical activity scores. Moreover, a bias could be caused by the fact that in patients with UC a surveillance colonoscopy was initiated without consideration of the FC finding. Another important limitation of the study is that we have not measured all of the variables the clinicians may consider when making their decision. It is a fact that further diagnostic evaluation or drug modifications are effected by multiple factors not just FC measurements. Moreover, with regard to the retrospective study design, the exact impact of FC measurement on clinical decisions remains unclear. Therefore, further prospective studies are needed to clarify this question unambiguously. For example, it would be useful to survey the physician’s directly about the influence of FC measurement in their overall clinical decision-making.

In conclusion, FC may be a parameter that influences physician’s clinical decision-making in IBD patients beside other clinical and diagnostic variables. Significantly more clinical consequences were detected in the case cohort compared to the control cohort. In addition, in patients with FC values > 250 mg/Kg more clinical consequences were detected than in patients with FC values ≤ 250 mg/Kg. The influence of FC on decision-making appeared to be stronger in patients with UC than in patients with CD, but other factors such as age, gender and laboratory findings also appear to have an important impact. For this reason the evaluation of FC in the patient’s clinical context is therefore important. The introduction of routine point-of-care FC measurement may improve the appropriateness of clinical decision-making, reduce adverse events associated with injudicious use of medications or invasive procedures, and reduce costs.

## Supporting information

S1 Fig A and B shows the results of FC level with regard to gender and disease activity status as box plots. C, D and are showing Spearman correlations between FC level and age, FC level and CRP level as well as FC level and platelet count.
(TIF)

## References

[1] Rogler G, Aldeguer X, Kruis W, Lasson A, Mittmann U, Nally K, et al Concept for a rapid point-of-care calprotectin diagnostic test for diagnosis and disease activity monitoring in patients with inflammatory bowel disease: Expert clinical opinion. J Crohns Colitis. 2013;7:670–7. 10.1016/j.crohns.2013.02.014 23517932

[2] Heida A, Park KT, van Rheenen PF. Clinical Utility of Fecal Calprotectin Monitoring in Asymptomatic Patients with Inflammatory Bowel Disease: A Systematic Review and Practical Guide. Inflamm Bowel Dis. 2017;23:894–902. 10.1097/MIB.0000000000001082 28511198 PMC5434712

[3] Carter MJ, Lobo AJ, Travis SPL. Guidelines for the management of inflammatory bowel disease in adults. Gut. 2004;53:V1–V16. 10.1136/gut.2004.043372 15306569 PMC1867788

[4] Lin JF, Chen JM, Zuo JH, Yu A, Xiao ZJ, Deng FH, et al Meta-analysis: Fecal Calprotectin for Assessment of Inflammatory Bowel Disease Activity. Inflamm Bowel Dis. 2014;20:1407–15. 10.1097/MIB.0000000000000057 24983982

[5] Dignass A, Eliakim R, Magro F, Maaser C, Chowers Y, Geboes K, et al Second European evidence-based consensus on the diagnosis and management of ulcerative colitis Part 1: Definitions and diagnosis. J Crohns Colitis. 2012;6:965–90. 10.1016/j.crohns.2012.09.003 23040452

[6] Van Assche G, Dignass A, Panes J, Beaugerie L, Karagiannis J, Allez M, et al The second European evidence-based Consensus on the diagnosis and management of Crohn's disease: Definitions and diagnosis. J Crohns Colitis. 2010;4:7–27. 10.1016/j.crohns.2009.12.003 21122488

[7] Rokkas T, Portincasa P, Koutroubakis IE. Fecal Calprotectin in Assessing Inflammatory Bowel Disease Endoscopic Activity: a Diagnostic Accuracy Meta-analysis. J Gastrointestin Liver Dis. 2018;27:299–306. 10.15403/jgld.2014.1121.273.pti 30240474

[8] Smith LA, Gaya DR. Utility of faecal calprotectin analysis in adult inflammatory bowel disease. World J Gastroenterol. 2012;18:6782–9. 10.3748/wjg.v18.i46.6782 23239916 PMC3520167

[9] Mumolo MG, Bertani L, Ceccarelli L, Laino G, Di Fluri G, Albano E, et al From bench to bedside: Fecal calprotectin in inflammatory bowel diseases clinical setting. World J Gastroenterol. 2018;24:3681–94. 10.3748/wjg.v24.i33.3681 30197475 PMC6127662

[10] Røseth AG, Schmidt PN, Fagerhol MK. Correlation between Faecal Excretion of Indium-111-Labelled Granulocytes and Calprotectin, a Granulocyte Marker Protein, in Patients with Inflammatory Bowel Disease. Scand J Gastroenterol. 1999;34:50–4. 10.1080/00365529950172835 10048733

[11] Motaganahalli S, Beswick L, Con D, van Langenberg DR. Faecal calprotectin delivers on convenience, cost reduction and clinical decision making in inflammatory bowel disease: a real world cohort study. Intern Med J. 2019;49:94–100. 10.1111/imj.14027 29962008

[12] Naismith GD, Smith LA, Barry SJE, Munro JI, Laird S, Rankin K, et al A prospective single-centre evaluation of the intra-individual variability of faecal calprotectin in quiescent Crohn's disease. Aliment Pharmacol Ther. 2013;37:613–21. 10.1111/apt.12221 23347334

[13] Langhorst J, Elsenbruch S, Koelzer J, Rueffer A, Michalsen A, Dobos GJ. Noninvasive markers in the assessment of intestinal inflammation in inflammatory bowel diseases: performance of fecal lactoferrin, calprotectin, and PMN-elastase, CRP, and clinical indices. Am J Gastroenterol. 2008;103:162–9. 10.1111/j.1572-0241.2007.01556.x 17916108

[14] Chen JM, Liu T, Gao S, Tong XD, Deng FH, Nie B. Efficacy of noninvasive evaluations in monitoring inflammatory bowel disease activity: A prospective study in China. World J Gastroenterol. 2017;23:8235–47. 10.3748/wjg.v23.i46.8235 29290660 PMC5739930

[15] Xiang JY, Ouyang Q, Li GD, Xiao NP. Clinical value of fecal calprotectin in determining disease activity of ulcerative colitis. World J Gastroenterol. 2008;14:53–7. 10.3748/wjg.14.53 18176961 PMC2673391

[16] D'Haens G, Ferrante M, Vermeire S, Baert F, Noman M, Moortgat L, et al Fecal Calprotectin is a Surrogate Marker for Endoscopic Lesions in Inflammatory Bowel Disease. Inflamm Bowel Dis. 2012;18:2218–24. 10.1002/ibd.22917 22344983

[17] Lin WC, Wong JM, Tung CC, Lin CP, Chou JW, Wang HY, et al Fecal calprotectin correlated with endoscopic remission for Asian inflammatory bowel disease patients. World J Gastroenterol. 2015;21:13566–73. 10.3748/wjg.v21.i48.13566 26730169 PMC4690187

[18] Zittan E, Kelly OB, Gralnek IM, Silverberg MS, Hillary Steinhart A. Fecal calprotectin correlates with active colonic inflammatory bowel disease but not with small intestinal Crohn's disease activity. JGH Open. 2018;2:201–6. 10.1002/jgh3.12068 30483590 PMC6207015

[19] Mosli MH, Zou G, Garg SK, Feagan SG, MacDonald JK, Chande N, et al C-Reactive Protein, Fecal Calprotectin, and Stool Lactoferrin for Detection of Endoscopic Activity in Symptomatic Inflammatory Bowel Disease Patients: A Systematic Review and Meta-Analysis. Am J Gastroenterol. 2015;110:802–819. 10.1038/ajg.2015.120 25964225

[20] Costa F, Mumolo MG, Ceccarelli L, Bellini M, Romano MR, Sterpi C, et al Calprotectin is a stronger predictive marker of relapse in ulcerative colitis than in Crohn's disease. Gut. 2005;54:364–8. 10.1136/gut.2004.043406 15710984 PMC1774401

[21] IBD Clinic. Modified Harvey Bradshaw Index Assessment for Crohn’s Disease Activity. http://www.ibdclinic.ca/media/uploads/harvey_bradshaw_index_09_2016.pdf Cited 15.01.2019.

[22] IBD Clinic. Partial Mayo Scoring Index Assessment for Ulcerative Colitis Activity. http://www.ibdclinic.ca/media/uploads/partial_mayo_09_2016.pdf Cited 15.01.2019.

[23] Silverberg MS, Satsangi J, Ahmad T, Arnott I, Bernstein CN, Brant SR, et al Toward an Integrated Clinical, Molecular and Serological Classification of Inflammatory Bowel Disease: Report of a Working Party of the 2005 Montreal World Congress of Gastroenterology. Can J Gastroenterol. 2005;19:5A–36A. 10.1155/2005/269076 16151544

[24] Huang VW, Prosser C, Kroeker KI, Wang H, Shalapay C, Dhami N, et al Knowledge of Fecal Calprotectin and Infliximab Trough Levels Alters Clinical Decision-making for IBD Outpatients on Maintenance Infliximab Therapy. Inflamm Bowel Dis. 2015;21:1359–67. 10.1097/MIB.0000000000000376 25989340 PMC4450916

[25] Rosenfeld G, Greenup AJ, Round A, Takach O, Halparin L, Saadeddin A, et al FOCUS: Future of fecal calprotectin utility study in inflammatory bowel disease. World J Gastroenterol. 2016;22:8211–8. 10.3748/wjg.v22.i36.8211 27688663 PMC5037090

[26] Abej E, El-Matary W, Singh H, Bernstein CN. The Utility of Fecal Calprotectin in the Real-World Clinical Care of Patients with Inflammatory Bowel Disease. Can J Gastroenterol Hepatol. 2016;2016:2016:2483261 10.1155/2016/2483261 27774443 PMC5059522

[27] El-Matary W, Abej E, Deora V, Singh H, Bernstein CN. Impact of Fecal Calprotectin Measurement on Decision-making in Children with Inflammatory Bowel Disease. Front Pediatr. 2017;5:7 10.3389/fped.2017.00007 28180127 PMC5263122

[28] Derwa Y, Williams CJM, Sood R, Mumtaz S, Bholah MH, Selinger CP, et al Factors affecting clinical decision-making in inflammatory bowel disease and the role of point-of-care calprotectin. Therap Adv Gastroenterol. 2018;11:1–18. 10.1177/1756283X17744739 29383026 PMC5784497

[29] Peyrin-Biroulet L, Sandborn W, Sands BE, Reinisch W, Bemelman W, Bryant RV, et al Selecting Therapeutic Targets in Inflammatory Bowel Disease (STRIDE): Determining Therapeutic Goals for Treat-to-Target. Am J Gastroenterol. 2015;110:1324–38. 10.1038/ajg.2015.233 26303131

[30] Vermeire S, Van Assche G, Rutgeerts P. The role of C-reactive protein as an inflammatory marker in gastrointestinal diseases. Nat Clin Pract Gastroenterol Hepatol. 2005;2:580–6. 10.1038/ncpgasthep0359 16327837

[31] Saverymuttu SH, Hodgson HJF, Chadwick VS, Pepys MB. Differing acute phase responses in Crohn's disease and ulcerative colitis. Gut. 1986;27:809–13. 10.1136/gut.27.7.809 3732890 PMC1433572

[32] Peyrin-Biroulet L, Panés J, Sandborn WJ, Vermeire S, Danese S, Feagan BG, et al Defining Disease Severity in Inflammatory Bowel Diseases: Current and Future Directions. Clin Gastroenterol Hepatol. 2016;14:348–354. 10.1016/j.cgh.2015.06.001 26071941

[33] Panés J, Bouzas R, Chaparro M, García-Sánchez V, Gisbert JP, Martínez de Guereñu B, et al Systematic review: the use of ultrasonography, computed tomography and magnetic resonance imaging for the diagnosis, assessment of activity and abdominal complications of Crohn's disease. Aliment Pharmacol Ther. 2011;34:125–45. 10.1111/j.1365-2036.2011.04710.x 21615440

[34] Galgut BJ, Lemberg DA, Day AS, Leach ST. The Value of Fecal Markers in Predicting Relapse in Inflammatory Bowel Diseases. Front Pediatr. 2018;5:292 10.3389/fped.2017.00292 29404311 PMC5780398

[35] Wright EK, Kamm MA, De Cruz P, Hamilton AL, Ritchie KJ, Krejany EO, et al Measurement of Fecal Calprotectin Improves Monitoring and Detection of Recurrence of Crohn's Disease After Surgery. Gastroenterology. 2015;148:938–947. 10.1053/j.gastro.2015.01.026 25620670

[36] Colombel JF, Panaccione R, Bossuyt P, Lukas M, Baert F, Vaňásek T, et al Effect of tight control management on Crohn's disease (CALM): a multicentre, randomised, controlled phase 3 trial. Lancet. 2017;390:2779–89. 10.1016/S0140-6736(17)32641-7 29096949

[37] Parente F, Molteni M, Marino B, Colli A, Ardizzone S, Greco S, et al Are Colonoscopy and Bowel Ultrasound Useful for Assessing Response to Short-Term Therapy and Predicting Disease Outcome of Moderate-to-Severe Forms of Ulcerative Colitis?: A Prospective Study. Am J Gastroenterol. 2010;105:1150–7. 10.1038/ajg.2009.672 19997096

[38] De Vos M, Louis EJ, Jahnsen J, Vandervoort JGP, Noman M, Dewit O, et al Consecutive fecal calprotectin measurements to predict relapse in patients with ulcerative colitis receiving infliximab maintenance therapy. Inflamm Bowel Dis. 2013;19:2111–7. 10.1097/MIB.0b013e31829b2a37 23883959

[39] Prager M, Büning C. Klinik, CRP, Calprotectin, MRT oder Endoskopie? Strategien zur sinnvollen Therapieüberwachung bei CED. coloproctology. 2014;36:250–8. 10.1007/s00053-014-0464-7

[40] Lee SH, Kim MJ, Chang K, Song EM, Hwang SW, Park SH, et al Fecal calprotectin predicts complete mucosal healing and better correlates with the ulcerative colitis endoscopic index of severity than with the Mayo endoscopic subscore in patients with ulcerative colitis. BMC Gastroenterol. 2017;17:110 10.1186/s12876-017-0669-7 29061121 PMC5654142

[41] Sandborn WJ, Panés J, Zhang H, Yu D, Niezychowski W, Su C. Correlation Between Concentrations of Fecal Calprotectin and Outcomes of Patients With Ulcerative Colitis in a Phase 2 Trial. Gastroenterology. 2016;150:96–102. 10.1053/j.gastro.2015.09.001 26376350

