# Supplementary figures and images for: Impact of faecal calprotectin measurement on clinical decision-making in patients with Crohn’s disease and ulcerative colitis
Source: PLoS One. 2019 Oct 24;14(10):e0223893. doi: 10.1371/journal.pone.0223893 (PMC6812761; doi:10.1371/journal.pone.0223893)

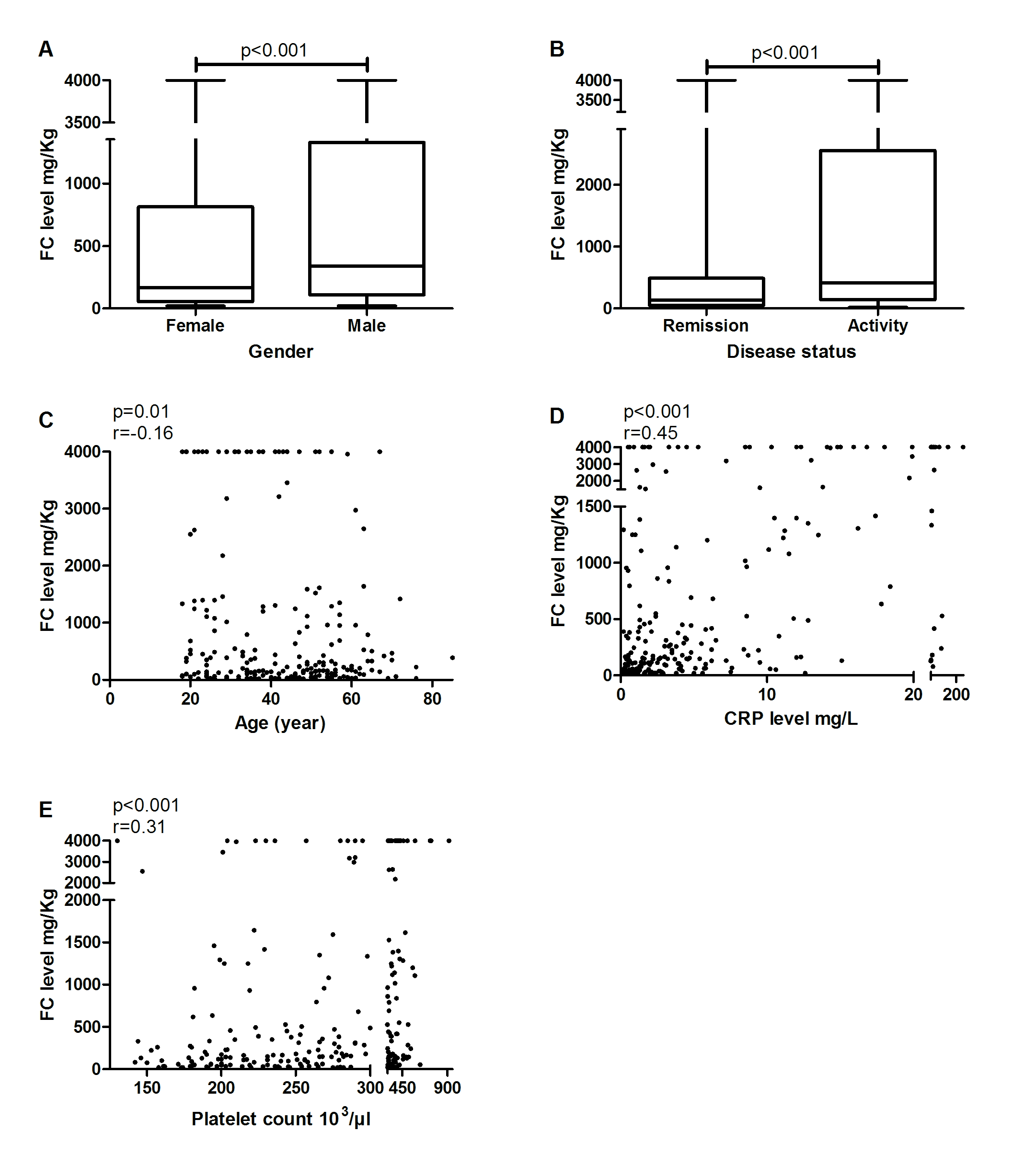

Supplement: S1 Fig — A and B shows the results of FC level with regard to gender and disease activity status as box plots. C, D and are showing Spearman correlations between FC level and age, FC level and CRP level as well as FC level and platelet count. (TIF) [file pone.0223893.s001.tif]
